# Development and Validation of an LC-MS/MS Method for Quantification of the Novel Antibacterial Candidate DA-7010 in Plasma and Application to a Preclinical Pharmacokinetic Study

**DOI:** 10.3390/ph14020163

**Published:** 2021-02-18

**Authors:** Mi Hye Kwon, Dae Young Lee, Hee Eun Kang

**Affiliations:** 1 College of Pharmacy and Integrated Research Institute of Pharmaceutical Sciences, The Catholic University of Korea, Bucheon 14662, Korea; kwonmh@kirams.re.kr; 2 Korea Institute of Radiological & Medical Sciences, Seoul 01812, Korea; 3 Research Center, Dong-A ST Co. Ltd., Yongin 17073, Korea; dylee@donga.co.kr

**Keywords:** DA-7010, LC-MS/MS, mouse, rat, dog, plasma, pharmacokinetics

## Abstract

DA-7010 is a new candidate for an antibacterial agent that targets Gram-negative pathogens by acting as a leucyl-tRNA synthetase inhibitor. In this study, a simple and rapid liquid chromatography tandem mass spectrometry (LC-MS/MS) method was developed to determine DA-7010 levels in the plasma from mice, rats, and dogs. Plasma samples were mixed with methanol for protein precipitation. Chromatographic separation was carried out using a reversed-phase C_18_ column (Agilent Poroshell 120, 50 × 3.0 mm, 2.7 μm). An isocratic elution of the mobile phase consisting of 5 mM formic acid in water and acetonitrile at a ratio of 84:16 (*v*/*v*) was applied at a flow rate of 0.3 mL/min. The total chromatographic run time was 3.5 min. Multiple reaction monitoring (MRM) mode was used for mass spectrometric detection using an Agilent 6460 triple quadrupole coupled with an electrospray ionization (ESI) source operated in positive-ion mode. The MRM transitions analyzed were *m/z* 220.1→162.1 for DA-7010 and *m/z* 206.1→170.1 for the internal standard (structural analogue of DA-7010). Calibration curves were constructed in the range of 10–10,000 ng/mL. The intra- and interday precision and accuracy were within 11.3%, excluding those for the lower limit of quantification (LLOQ) samples, which were within 17.1%. The developed LC-MS/MS method was successfully validated and applied in preclinical pharmacokinetic studies of DA-7010 in mice, rats, and dogs following single oral administrations. The oral absorption of DA-7010 was rapid, and the systemic exposure was approximately four times higher in the dogs than in the mice or rats.

## 1. Introduction

**Figure 1:**
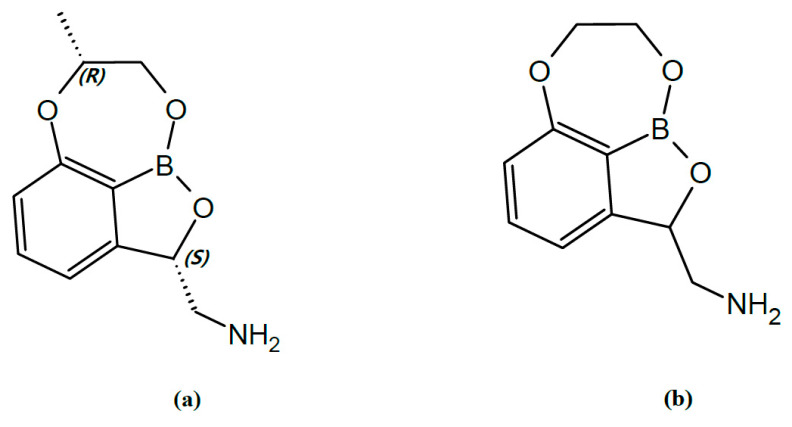
Chemical structures of DA-7010 and the internal standard (IS): (**a**) DA-7010; (**b**) the IS.

Infections of carbapenem-resistant or multidrug-resistant Gram-negative pathogens are major healthcare concerns due to high morbidity and the absence of new drug development [1]. DA-7010 (((2S,7R)-7-methyl-7,8-dihydro-2H-1,6,9-trioxa-9a-borabenzo[cd]azulen-2-yl)methanamine; Figure 1a; confirmed by HR-MS and ^1^H-NMR spectra (Figure S1)) is a new candidate for an antibacterial agent against Gram-negative pathogens due to acting as a leucyl-tRNA synthetase (LeuRS) inhibitor with a tricyclic benzoxaborole moiety [2,3]. In the past decade, benzoxaborole moieties have been applied in the design of various compounds for antimicrobial and anti-inflammatory agents due to their drugability [4]. A subset of benzoxaboroles inhibit LeuRS through an oxaborole tRNA-trapping mechanism, which forms a stable leucyl-tRNA-benzoxaborole adduct [5]. Because the structures of aminoacyl-tRNA synthetases (ARSs) found in eukaryotes have considerable structural differences from those in prokaryotes, it is possible to develop highly selective inhibitors that can achieve antibacterial-specific responses [6]. Apart from translation, emerging roles of host ARSs have been identified in anti-infectious signaling and antimicrobial immunity [7,8]. In recent years, basic and clinical studies have been underway to apply therapeutics based on ARS inhibition to various diseases such as neurological disorders, fibrosis, and cancer as well as infectious diseases [9,10,11]. 

DA-7010 showed excellent in vitro and in vivo antibacterial efficacy, with a minimum inhibitory concentration (MIC) of 0.25–2 μg/mL for DA-7010 against Gram-negative bacteria (except for *Pseudomonas aeruginosa*) and effective oral doses of 1.9–3 mg/kg that protected 50% of mice systemically infected with carbapenem-resistant *Acinetobacter baumannii* (ED_50_) [2,3]. Pharmacokinetic studies of DA-7010 in experimental animals are essential for the further development of DA-7010 as an antibacterial agent. The preclinical pharmacokinetic characterization of DA-7010 is required for a comprehensive understanding of its efficacy and potential toxicity. For pharmacokinetic studies of DA-7010 to be successful in various species of animals, efficient and robust quantitative methods for determining DA-7010 in biological fluids are essential.

In this study, a simple LC-MS/MS method for the robust and accurate determination of DA-7010 in plasma from mice, rats, and dogs was developed and validated. The method was optimized to realize the precise and accurate quantification of DA-7010 in plasma samples from various animal species. Pharmacokinetic studies of DA-7010 following a single oral administration to mice, rats, and dogs were performed using the proposed method.

## 2. Results and Discussion

### 2.1. Development of Analytical Method 

**Figure 2:**
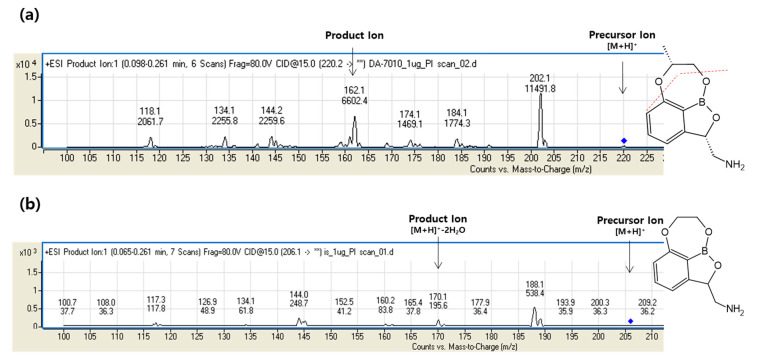
Product ion mass spectra of (**a**) DA-7010 and (**b**) the IS.

We optimized the instrumental parameters for mass spectrometry to obtain sensitive and robust signals for the analyte. The internal standard (IS) for the DA-7010 analysis, 7,8-dihydro-2H-1,6,9-trioxa-9a-borabenzo[cd]azulen-2-yl)methanamine (a DA-7010 analogue; Figure 1b), was chosen for its structural similarity to DA-7010. Full-scan mass spectra (Figure S2) and product ion spectra of DA-7010 and the IS (Figure 2) were obtained in positive electrospray ionization (ESI) mode. For the quantification of DA-7010, one selected precursor-to-product ion transition of *m/z* 220.1 ([M+H]^+^)→162.1 (loss of C_3_H_6_O) was monitored. The transition selected and monitored for the IS was *m/z* 206.1 ([M+H]^+^)→170.1 (loss of 2H_2_O).

The chromatographic conditions were optimized with a reversed-phase HPLC Poroshell 120 C_18_ column (50 mm L. × 3.0 mm i.d.; particle size, 2.7 μm) that provided excellent resolution and peak symmetry. An isocratic elution of 5 mM formic acid in water (A) and acetonitrile (B) (A:B = 84:16 (*v*/*v*)) resulted in optimum retention times for the analyte and IS and the avoidance of the matrix effect for DA-7010. An acceptable peak shape and resolution along with an adequate retention time were achieved for the analyte in this optimized chromatographic condition. The total chromatographic run time for each was 3.5 min, which was highly efficient. The retention times of DA-7010 and the IS were approximately 1.8 and 1.2 min, respectively. 

Protein precipitation methods were initially attempted for sample preparation because of their simplicity. Sufficient and reproducible recoveries of DA-7010 and the IS were achievable by simple protein precipitation using methanol.

### 2.2. Validation of Analytical Method 

#### 2.2.1. Selectivity and Carryover

**Figure 3:**
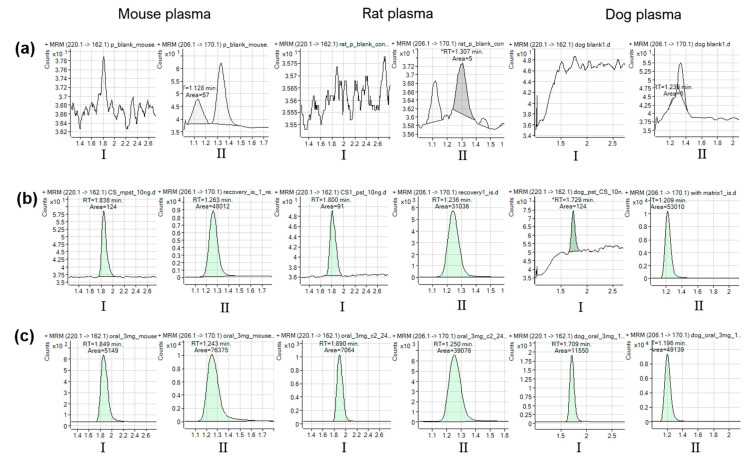
Representative multiple reaction monitoring (MRM) chromatograms of DA-7010 (I) and the IS (II) in each mouse, rat, and dog plasma sample: (**a**) blank plasma; (**b**) blank plasma spiked with lower limit of quantification (LLOQ) level of DA-7010 (10 ng/mL) or mixed with the IS at working concentrations (10 μg/mL); (**c**) plasma samples collected 4 h after oral administration of 3 mg/kg DA-7010 to mouse, rat, and dog with determined DA-7010 concentrations of 334, 648, and 1952 ng/mL, respectively.

Representative chromatograms of blank mouse, rat, and dog plasma are presented (Figure 3a), as well as plasma spiked with DA-7010 at the lower limit of quantification (LLOQ) level and the IS (Figure 3b). The chromatograms for plasma samples obtained 4 h after the oral administration of DA-7010 are presented in Figure 3c. No endogenous interference was observed at the retention times for DA-7010 or the IS, indicating that the developed method was specific. No carryover was found under the conditions used, as observable by the absence of a significant peak (≥20% of the LLOQ) of DA-7010 in a blank plasma sample analyzed right after the injection of a high quality control (QC) sample.

#### 2.2.2. Linearity and Sensitivity

Calibration curves were generated using a weighted (1/x or 1/x^2^) least-squares linear regression plot of the peak area ratio of DA-7010 to the IS (y) versus the relative concentration of DA-7010 to the IS (x). The linearity of the calibration curves was observed over the DA-7010 concentration range of 10‒10,000 ng/mL (Figure S3). The correlation coefficients (*r*^2^) of the calibration curves were > 0.995. The typical regression equations of the calibration curves were as follows: y = 1.5989x + 0.0002938 (*r*^2^ = 0.9992) for the mouse plasma samples; y = 2.7906x − 0.0001545 (*r*^2^ = 0.9992) for the rat plasma samples; y = 1.2445x + 0.00001202 (*r*^2^ = 0.9951) for the dog plasma samples. The deviations of the back-calculated concentrations of the calibration samples from the nominal values were within the acceptable range (<15% except for the LLOQ level, <20%). The LLOQ for DA-7010 was confirmed as 10 ng/mL with a good signal-to-noise ratio (>10) and acceptable accuracy (80‒120%) and precision (relative standard deviation (RSD) < 20%). The developed method had enough sensitivity for determining DA-7010 plasma levels.

#### 2.2.3. Precision and Accuracy

The intra- and interday precisions and accuracies for the analyses of QC samples with LLOQ, low, medium, and high concentrations of DA-7010 in mouse, rat, and dog plasma are presented in Table 1. The analytical precisions (expressed as RSDs) for the intra- and interday analyses were within 11.3%, except for the LLOQ (RSD < 17.1%). The intra- and interday accuracies for the DA-7010 analyses in plasma from various species were 89.8–106%, including for the LLOQ. These results demonstrate that the developed method had sufficient precision and accuracy for the quantification of DA-7010 in plasma from mice, rats, and dogs.

#### 2.2.4. Recovery and Matrix Effect

The plasma samples were treated with methanol to precipitate the proteins and to achieve a reproducible recovery of DA-7010. The recovery and matrix effect are summarized in Table 2. The average recoveries of DA-7010 in the mouse, rat, and dog plasma after sample preparation were 86.2‒93.6%, 69.5‒80.2%, and 74.3‒83.2%, respectively, and those for the IS were 87.4%, 84.9%, and 93.5%, respectively. Both DA-7010 and the IS showed consistent and reproducible recoveries (RSD < 13.8%) from the plasma of all the tested species. The matrix effects of DA-7010 were within the range of 91.4‒119%, and the RSDs (%) from the six different plasma sources were < 8.04%. These results indicate that interferences from plasma matrix were adequately eliminated through proper sample preparation and chromatographic separation. Although matrix effects in the IS were observed in the mouse and rat plasma, an RSD (%) < 4.20 based on six different donors from each species ensured the robustness of the analytical method.

#### 2.2.5. Stability of the Analyte

Table 3 summarizes the stability of DA-7010 in the plasma from each species evaluated by analyzing three replicates of QC samples (low, medium, and high concentrations) under various storage conditions. DA-7010 was stable (mean recoveries > 85.5%) for at least 8 h at room temperature, 24 h when kept in an LC vial at 4 °C after sample preparation, and 1 month when stored at –20 °C. The analyte was stable even after three freeze/thaw (–20 °C/room temperature) cycles (mean recoveries > 87.7%). Therefore, DA-7010 is stable during sample storage, preparation, and analysis (in the plasma of the species analyzed). 

### 2.3. Application in Pharmacokinetic Studies

**Figure 4:**
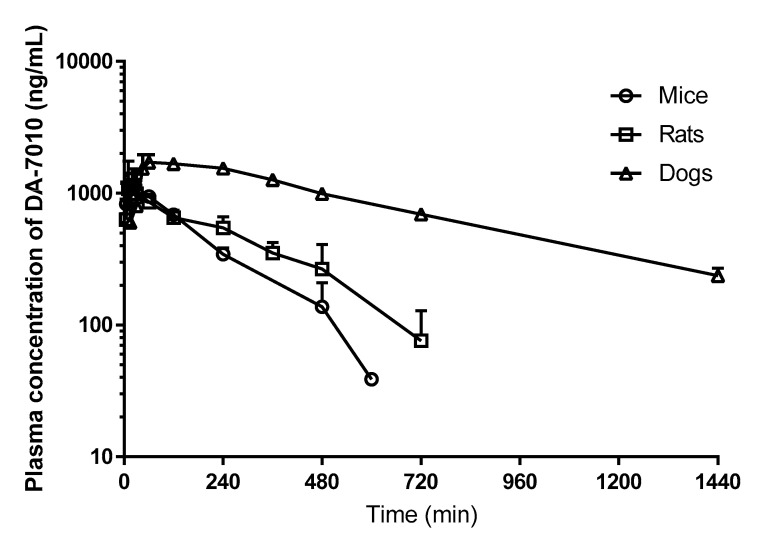
Mean arterial plasma concentration–time profiles of DA-7010 following oral administration of DA-7010 at a dose of 3 mg/kg to mice (*n* = 6 for each point), rats (*n* = 7), and dogs (*n* = 4). Data are expressed as mean ± SD.

The validated LC-MS/MS method was applied to pharmacokinetic studies of DA-7010 after administration of a single oral dose (3 mg/kg) of DA-7010 to mice, rats, and dogs. It should be noted that this oral dose has been found to be an ED_50_ in mice with systemic infection. The profiles of the mean plasma concentration of DA-7010 versus time are presented in Figure 4. The developed method proved to have enough sensitivity for the quantification of DA-7010 for up to 10, 12, and 24 h in the mouse, rat, and dog plasma samples, respectively. The pharmacokinetic parameters of DA-7010 in the mice, rats, and dogs are summarized in Table 4. The total area under the plasma concentration–time curve from time zero to infinity (AUC_0-∞_) of DA-7010 in the dogs was much greater than those in the mice or rats. The maximum plasma concentration (*C*_max_) values for the studied animal species were similar to the reported MICs for DA-7010, 0.25–2 μg/mL [2,3]. In the mice and rats, the absorption of DA-7010 was rapid in view of the fast time to reach *C*_max_ (*T*_max_). Considering the dissolution time for DA-7010 in the gelatin capsule, it was also rapidly absorbed in dogs. 

## 3. Materials and Methods

### 3.1. Chemicals and Reagents

The DA-7010 hydrochloride (purity, 99.5%; batch no. R-K-14001) and the IS for the DA-7010 analysis (purity, 96.4%) were products of Dong-A ST (Yongin, South Korea). The other solvents and chemicals were either HPLC or reagent grade. 

### 3.2. Instrumental Conditions for LC-MS/MS 

The instrumental system for LC-MS/MS consisted of an Agilent 1260 LC system and an Agilent 6460 triple quadrupole tandem mass spectrometer (Agilent, Waldbronn, Germany). The instrument control, data acquisition, and processing of the data were conducted using the Agilent Mass Hunter Workstation software (ver. B.04.01). 

A reversed-phase HPLC column (Agilent Poroshell 120 C_18_ column; 50 × 3.0 mm; particle size, 2.7 μm) was used for appropriate chromatographic separation. The isocratic elution of the mobile phase was accomplished with 5 mM formic acid in water and acetonitrile at a ratio of 84:16 (*v*/*v*) and a flow rate of 0.3 mL/min. To minimize contamination of the ESI source and mass spectrometer, the eluent was switched to a waste line during the first minute. The total run time was 3.5 min. The column and autosampler temperatures were set at 30 and 4 °C, respectively. 

The column eluent was monitored using a triple quadrupole tandem mass spectrometer equipped with an ESI source in positive ion mode. The instrumental conditions were as follows: gas temperature (350 °C), sheath gas temperature (400 °C), gas flow (13 L/min), sheath gas flow (12 L/min), nebulizer (55 psi), and electrospray voltage (3.5 kV). The fragmentor voltages for both DA-7010 and the IS were set at 80 V. The collision energies for DA-7010 and the IS were 13 and 15 eV, respectively. The precursor ([M + H]^+^)-to-product ion transitions used for quantification were *m/z* 220.1→162.1 for DA-7010 and *m/z* 206.1→170.1 for the IS.

### 3.3. Preparation of Calibration Standards and QC Samples

Standard stock solutions of DA-7010 and the IS were prepared by dissolving each drug in methanol to yield a nominal concentration of 1 mg/mL. Standard working solutions of DA-7010 were prepared by appropriate dilutions of the stock solutions with methanol (1–1000 μg/mL). The IS working solution was prepared at a concentration of 10 μg/mL from stock solution using methanol for dilution. 

Each working stock solution of DA-7010 (10 μL) was spiked into pooled, drug-free mouse, rat, or dog plasma (1000 μL) at final concentrations of 10, 25, 50, 250, 1000, and 10,000 ng/mL to prepare the calibration samples. The QC samples were prepared at final concentrations of 10 (LLOQ), 30 (low QC), 400 (medium QC), and 8000 (high QC) ng/mL in the same manner as the calibration samples by separately weighing the DA-7010. All the stock solutions and calibration and QC samples were immediately stored at −20 °C and brought to room temperature prior to use.

### 3.4. Sample Preparation

A 100 μL aliquot of the IS working solution (10 μg/mL in methanol) was added to each 25 μL plasma sample and mixed by vortexing to precipitate the protein. After centrifugation (16,000 *g*, 5 min), the supernatant was transferred to an LC vial, and a 2 μL aliquot was injected into the LC column for analysis.

### 3.5. Validation of Analytical Method 

The developed method has been validated according to the US FDA guidelines for the validation of bioanalytical methods [12] for selectivity, carryover, linearity, sensitivity, precision, accuracy, recovery, the matrix effect, and stability.

#### 3.5.1. Selectivity, Carryover, Linearity, and Sensitivity

To assess the selectivity of the method, 6 different sources of drug-free plasma from mice, rats, and dogs were analyzed for the presence of interfering peaks in the retention times of the analyte and the IS. To evaluate the carryover, a blank plasma sample was analyzed after injecting a high QC sample. The linearity was assessed based on calibration curves with 6 levels of the analyte as described in Section 3.3. The calibration curves were constructed using weighted (1/x or 1/x^2^) least-squares linear regression of the plot for the peak area ratio of DA-7010 to the IS (y) versus the relative concentration of DA-7010 to the IS (x). The analytical sensitivity was assessed by the LLOQ, which was defined as the lowest quantifiable level (signal-to-noise ratio > 10) with acceptable precision (RSD < 20%) and accuracy (80–120%).

#### 3.5.2. Precision and Accuracy

To determine the intraday precision and accuracy, 5 replicates of LLOQ, low, medium, and high QC samples were analyzed in a single run. The precision and accuracy for the interday analyses were assessed by analyzing QC samples at 4 levels for 5 consecutive days (more than 3 repetitions each time). The precision is expressed as the RSD (%), and the accuracy was calculated as (determined concentration/nominal concentration) × 100%. The acceptance criterion for the precision was set to RSD < 15% (< 20% for LLOQ), and that for the accuracy was 85–115% (80–120% for the LLOQ).

#### 3.5.3. Recovery and Matrix Effect

For the measurement of the recovery and matrix effects, 3 QC analyte levels and the IS were used. The peak areas of the analyte or IS in the extracted QC samples (spiked prior to extraction) were compared with those in the spiked samples (spiked into post-extracted blank plasma) with corresponding concentrations to determine the extraction recovery. The matrix effect was determined by comparing the peak areas of the analyte or IS from the spiked, post-extracted, blank plasma (with matrix) with those of the samples spiked with post-extracted water (without matrix).

#### 3.5.4. Stability of the Analyte

The stability of DA-7010 in the plasma from the mice, rats, and dogs was evaluated by analyzing 3 replicates of low, medium, and high QC samples under various storage conditions: the bench-top short-term stability (at room temperature for 8 h), post-preparative stability (prepared samples kept in LC vials at 4 °C for 24 h), freeze–thaw stability (3 freeze/thaw (−20 °C/room temperature) cycles), and long-term freezing stability (−20 °C for 1 month). The analyte was determined to be stable when the estimated recovery (determined concentration/nominal concentration × 100%) was in the range of 85–115% and the RSD was < 15%.

### 3.6. Pharmacokinetic Studies of DA-7010 in Mice, Rats, and Dogs

The validated method was applied to pharmacokinetic studies of DA-7010 in mice, rats, and dogs. The protocols for the animal study were approved by the Department of Laboratory Animals, Institutional Animal Care and Use Committee (IACUC), on the Sungsim Campus of the Catholic University of Korea (approval no. 2014-022) or by IACUC of Biotoxtech (Cheongju, South Korea) (approval no. 140765). Male ICR mice and Sprague-Dawley rats (~8 weeks old) were purchased from Samtaco Bio (Osan, South Korea). Male beagle dogs (~10 months old), which are products from Beijing Marshall Biotechnology (Beijing, China), were used for this study. The accommodation and handling procedures for the animals were similar to those previously reported [13,14]. Prior to dosing, the animals were fasted for 15 h and allowed to drink water freely. DA-7010 hydrochloride was administered in the pharmacokinetic study, and the dose of DA-7010 described below is based on the free base form of DA-7010.

DA-7010 (dissolved in distilled water) at a dose of 3 mg (in 10 mL) per kilogram was administered orally to the mouse groups A and B (*n* = 6 each) using gastric gavage. Blood samples (~ 50 μL) were collected into separate heparinized capillary tubes from the retro-orbital plexuses of mice that were under light anesthesia, which was achieved by isoflurane inhalation at 5, 30, 120, and 480 min for group A and 15, 60, 240, and 600 min for group B following the oral administration of DA-7010. The collected sample was immediately centrifuged, and a 25 μL plasma sample was stored at −20 °C until it was used for the LC-MS/MS analyses of DA-7010.

For the rat, the carotid artery was cannulated for serial blood sampling [14]. DA-7010 (dissolved in distilled water) at a dose of 3 mg (in 2 mL) per kilogram was administered orally to the rats (*n* = 7) using gastric gavage. Blood samples (~50 μL) were collected at 0, 5, 10, 20, 30, 60, 120, 240, 360, 480, and 720 min via the carotid artery following the oral administration of the drug. The cannula was flushed immediately after each blood sampling to prevent blood clotting using a 0.3 mL heparinized 0.9% NaCl-injectable solution (20 units/mL). The same procedure described above was used for the collection and storage of the plasma samples.

For the oral administration of DA-7010 in dogs, 3 mg/kg DA-7010 was loaded in a gelatin capsule (size 12, Torpac Inc., Fairfield, NJ, USA) without other excipients. Approximately 2 mL of blood was collected via the cephalic vein using a heparinized (100 IU/mL) 23 G syringe at pre-dose and 15, 30, 45, 60, 120, 240, 360, 480, 720, and 1440 min after the administration of the drug. The collection and storage of each plasma sample (25 μL) and extra plasma sample were performed using the same procedure described above. 

The pharmacokinetic parameters were calculated using noncompartmental analyses (WinNonlin; Pharsight Corporation, Mountain View, CA, USA) [15]. The AUC_0-∞_ was calculated using the trapezoidal rule-extrapolation method [16]. The *C*_max_ and *T*_max_ were read directly from the experimental data.

## 4. Conclusions

The fast and simple LC-MS/MS method developed in this study enables the robust and sensitive quantification of DA-7010 in plasma from mice, rats, and dogs. This method has proven to be accurate, precise, and highly efficient by avoiding the plasma matrix effects of various species and enabling the reproducible recovery of the analyte through simple protein precipitation. The method was successfully applied to pharmacokinetic studies of DA-7010 in these preclinical species following the oral administration of DA-7010. The oral absorption of DA-7010 was rapid, and the systemic exposure was approximately four times higher in dogs than in mice or rats. This is the first reported pharmacokinetic study of DA-7010, a new antibacterial drug candidate for Gram-negative pathogens. The analytical method and pharmacokinetic data presented in this study can be applied in further preclinical pharmacokinetic studies of DA-7010.

## Figures and Tables

**Table 1:** Intra- and interday precision and accuracy of analysis for DA-7010 in mouse, rat, and dog plasma.

Matrix	Nominal Concentration (ng/mL)	Intraday (*n* = 5)			Interday (*n* = 5)		
		Calculated Concentration (Mean, ng/mL)	Precision (RSD, %)	Accuracy (%)	Calculated Concentration (Mean, ng/mL)	Precision (RSD, %)	Accuracy (%)
Mouse plasma	10	9.23	14.9	92.3	9.55	10.8	95.5
	30	30.2	8.86	101	28.1	7.03	93.6
	400	394	7.89	98.4	372	4.91	93.1
	8000	7770	8.41	97.1	7760	11.3	97.0
Rat plasma	10	10.5	10.7	105	10.5	1.70	105
	30	28.4	3.90	94.6	27.2	3.31	90.5
	400	397	2.53	99.3	377	6.49	94.3
	8000	8590	1.82	107	8240	8.45	103
Dog plasma	10	10.4	17.1	104	10.6	4.82	106
	30	26.9	3.49	89.8	28.1	2.42	93.8
	400	369	8.08	92.2	370	3.51	92.5
	8000	7750	7.22	96.9	8330	6.76	104

**Table 2:** Extraction recovery and matrix effect of DA-7010 and the IS in mouse, rat, and dog plasma.

Matrix/Analytes	Nominal Concentration (ng/mL)	Recovery (*n* = 5)		Matrix Effect (*n* = 6)	
		Mean ± SD (%)	RSD (%)	Mean ± SD (%)	RSD (%)
Mouse plasma					
DA-7010	30	93.6 ± 5.99	6.40	95.5 ± 5.69	5.96
	400	86.2 ± 1.00	11.6	96.4 ± 7.75	8.04
	8000	88.9 ± 12.2	13.8	97.1 ± 3.50	3.61
IS	10,000	87.4 ± 1.22	1.40	71.1 ± 2.09	2.94
Rat plasma					
DA-7010	30	69.5 ± 1.88	2.72	103 ± 4.95	4.81
	400	69.7 ± 3.44	4.93	91.4 ± 4.67	5.11
	8000	80.2 ± 1.78	2.21	98.2 ± 2.45	2.50
IS	10,000	84.9 ± 2.49	2.93	50.8 ± 2.13	4.20
Dog plasma					
DA-7010	30	83.2 ± 4.96	5.96	119 ± 8.30	6.99
	400	74.5 ± 4.78	6.42	103 ± 3.87	3.76
	8000	74.3 ± 2.50	3.36	94.3 ± 2.32	2.46
IS	10,000	93.5 ± 4.18	4.47	95.7 ± 3.03	3.17

**Table 3:** Stability (% recovery, *n* = 3) of DA-7010 in mouse, rat, and dog plasma under various storage conditions.

Storage Conditions	Nominal Concentration (ng/mL)	Mouse Plasma		Rat Plasma		Dog Plasma	
		Mean ± SD (%)	RSD (%)	Mean ± SD (%)	RSD (%)	Mean ± SD (%)	RSD (%)
Short term ^1^	30	109 ± 4.97	4.54	110 ± 4.64	4.21	107 ± 3.08	2.88
	400	97.5 ± 6.48	6.65	97.9 ± 0.394	0.403	92.9 ± 1.62	1.74
	8000	89.0 ± 2.24	2.51	104 ± 0.647	0.623	108 ± 1.22	1.12
Post-preparative ^2^	30	106 ± 5.14	4.86	95.5 ± 3.65	3.82	110 ± 5.48	4.98
	400	92.7 ± 2.58	2.78	101 ± 1.33	1.32	108 ± 1.72	1.59
	8000	89.9 ± 7.84	8.73	111 ± 4.06	3.65	116 ± 2.34	2.02
Freeze–thaw ^3^	30	109 ± 6.63	6.10	107 ± 5.92	5.50	110 ± 3.32	3.01
	400	91.2 ± 2.22	2.43	94.8 ± 6.20	6.54	107 ± 2.01	1.89
	8000	87.7 ± 2.58	2.95	100 ± 0.407	0.406	114 ± 0.0718	0.0628
Long term ^4^	30	106 ± 1.54	1.46	85.5 ± 0.618	0.722	113 ± 0.500	0.442
	400	95.5 ± 3.04	3.19	95.2 ± 3.30	3.47	97.3 ± 1.22	1.25
	8000	88.5 ± 1.77	2.00	107 ± 2.67	2.49	111 ± 3.13	2.82

^1^ Storage at room temperature for 8 h; ^2^ Storage of prepared samples at 4 °C for 24 h; ^3^ Storage after 3 freeze/thaw (–20 °C/room temperature) cycles; ^4^ Storage at ‒20 °C over 1 month.

**Table 4:** Pharmacokinetic parameters for DA-7010 after single oral administration at a dose of 3 mg/kg to mice, rats, and dogs.

Parameters	Mice (*n* = 6 for Each Data Point) ^3^	Rats (*n* = 7)	Dogs (*n* = 4)
Body weight (kg)	0.0246 ± 0.000965	0.242 ± 0.00393	9.47 ± 0.738
AUC_0–∞_ (μg∙min/mL) ^1^	235	319 ± 45.5	1310 ± 95.7
Terminal half-life (min)	125	173 ± 48.6	452 ± 33.8
*C*_max_ (μg/mL)	1.28	1.33 ± 0.427	1.82 ± 0.0901
*T*_max_ (min) ^2^	15	20 (10‒240)	60 (45‒120)

^1^ Total area under the plasma concentration–time curve from time zero to infinity. ^2^ *T*_max_ is expressed as median (range). ^3^ Pharmacokinetic parameters were obtained from a mean concentration–time profile.

## Data Availability

The main part of the research data is contained in the article and Supplementary Materials. Other data including chromatograms are available from the corresponding author upon request.

## Supplementary Materials

The following are available online at https://www.mdpi.com/1424-8247/14/2/163/s1. Figure S1: HR-MS (A) and ^1^H-NMR (B) spectra of DA-7010, Figure S2: Full-scan mass spectra of DA-7010 (A) and the IS (B), Figure S3: Representative calibration curves with regression equations for DA-7010 in plasma from various species (mice (A), rats (B), and dogs (C)).
