# Supplementary material for: Development and Validation of an LC-MS/MS Method for Quantification of the Novel Antibacterial Candidate DA-7010 in Plasma and Application to a Preclinical Pharmacokinetic Study
Source: Pharmaceuticals (Basel). 2021 Feb 18;14(2):163. doi: 10.3390/ph14020163 (PMC7922803; doi:10.3390/ph14020163)

Figure S1. HR-MS (A) and ^{1}H-NMR (B) spectra of DA-7010. HR-MS spectrum was obtained in positive ESI mode using Thermo Scientific Q Exactive Orbitrap mass spectrometer. Accurate mass of $[M+H]^+$ ion of DA-7010 measured was consistent with calculated mass based on chemical formula of DA-7010 (220.11395 Da). ^{1}H-NMR spectrum of DA-7010 hydrochloride (in DMSO-*d*6) was obtained using Varian 400 MHz NMR spectrometer.

(A)

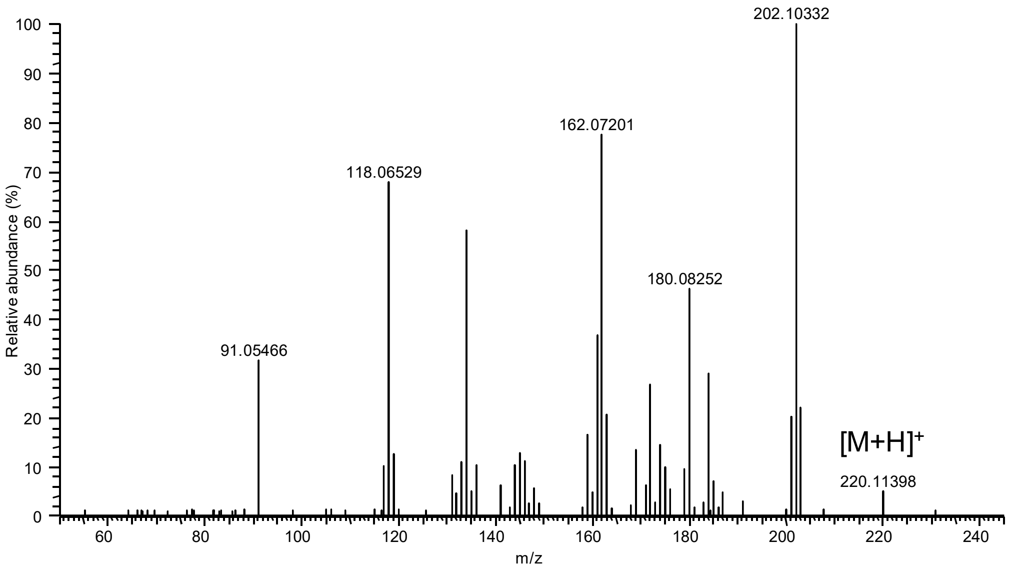

(B)

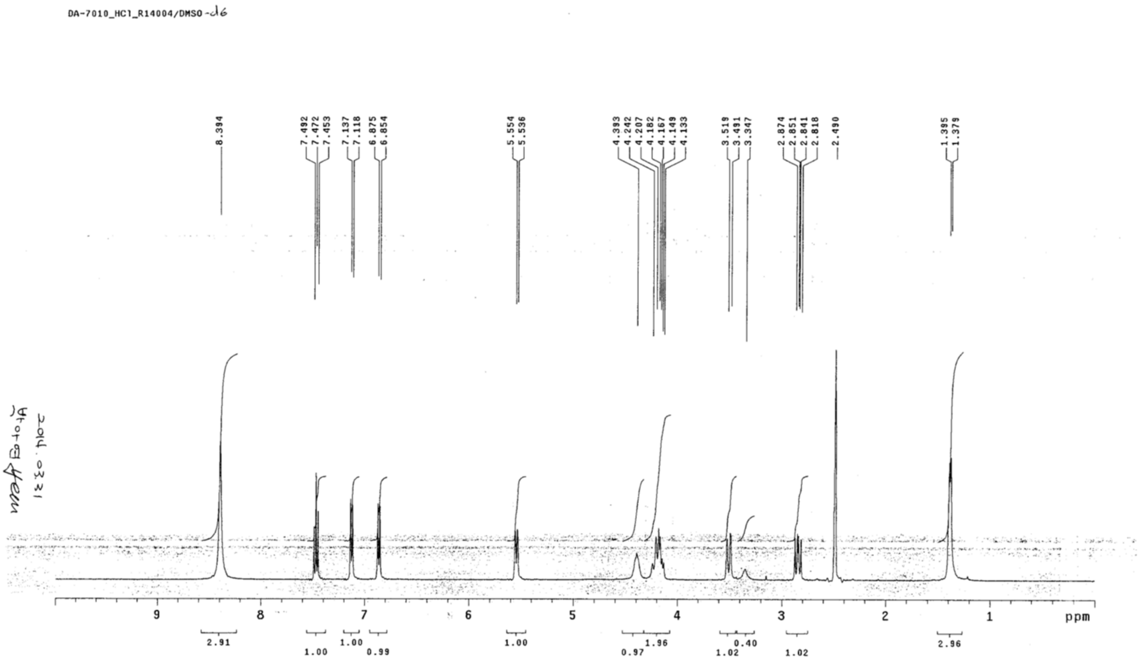

δ 1.41 (3H, d, *J* = 6.4 Hz), 2.87 (1H, dd, *J* = 13.2, 9.2 Hz), 3.52 (1H, dd, *J* = 13.2, 2.4 Hz), 4.15-4.26 (2H, m), 4.41 (1H, brs), 5.54-5.59 (1H, m), 6.88 (1H, *d, J* = 8.4 Hz), 7.14 (1H, *d, J* = 7.2 Hz), 7.49 (1H, *t, J* = 7.8 Hz), 8.40 (3H, brs)

Figure S2. Full-scan mass spectra of DA-7010 (A) and the IS (B). Spectra were obtained in MS2 scan mode (positive ESI with Fragment voltage of 135V) using an Agilent 6460 triple quadrupole tandem mass spectrometer. $[M+H]^+$ ions of DA-7010 and the IS were indicated by red arrows.

(A)

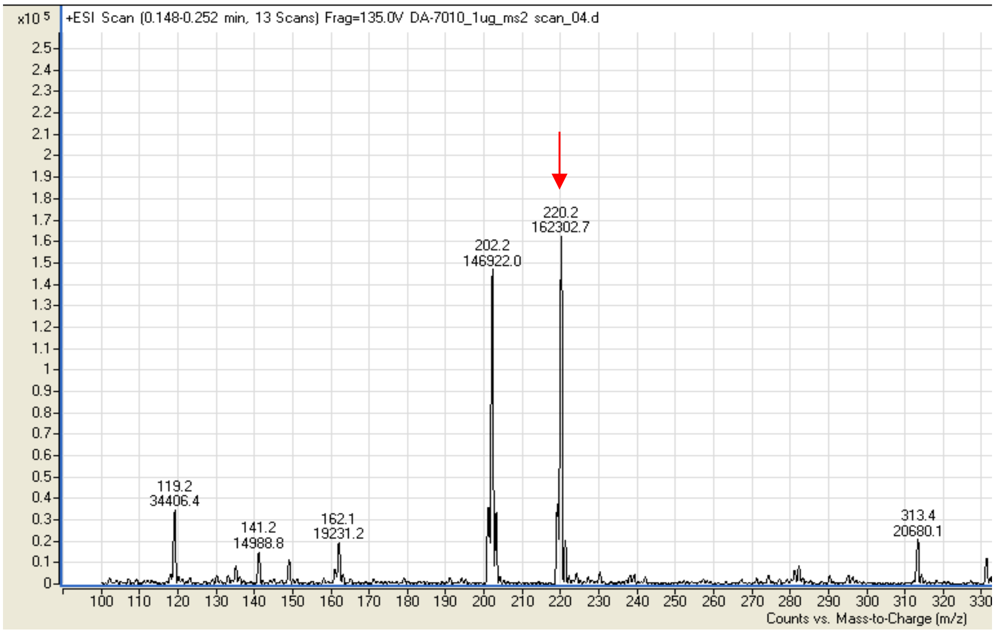

(B)

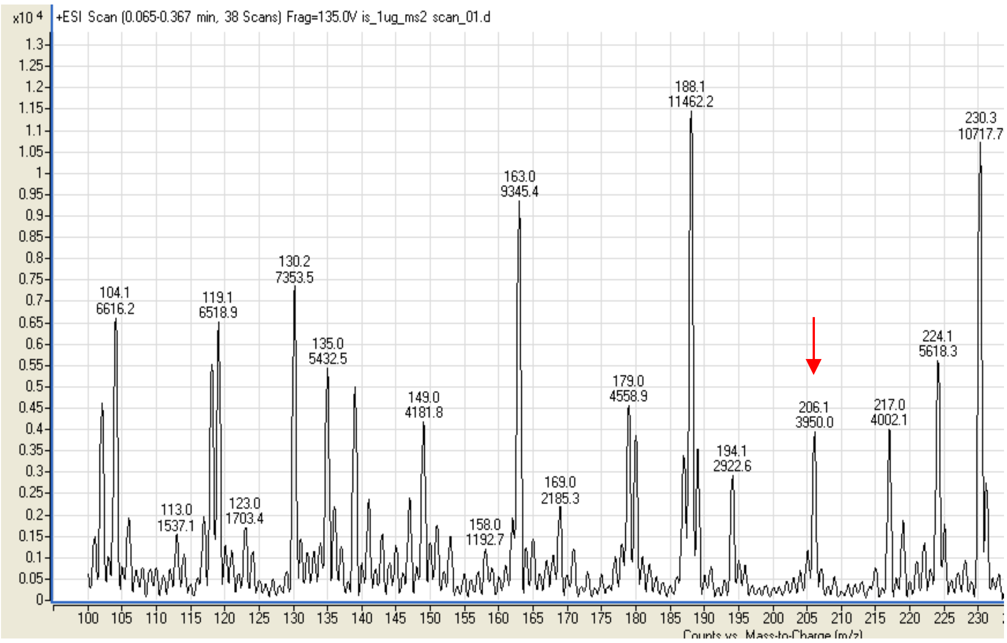

Figure S3. Representative calibration curves with regression equations for DA-7010 in plasma from various species (mice (A), rats (B) and dogs (C)). Calibration curves over the DA-7010 concentration range of 10–10000 ng/mL were constructed by plotting the peak area ratio of DA-7010 to the IS (y) versus the relative concentration of DA-7010 to the IS (x) with weighted ($1/x$ or $1/x^2$) least-squares linear regression.

(A)

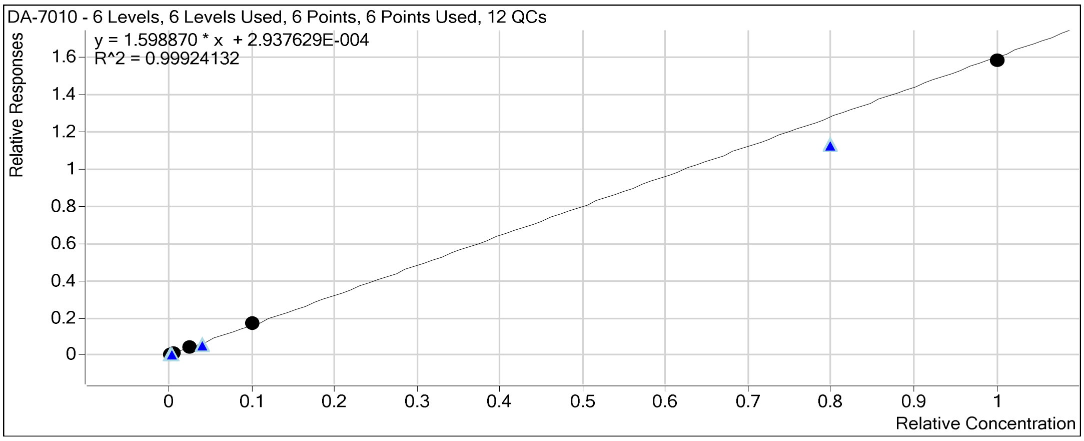

(B)

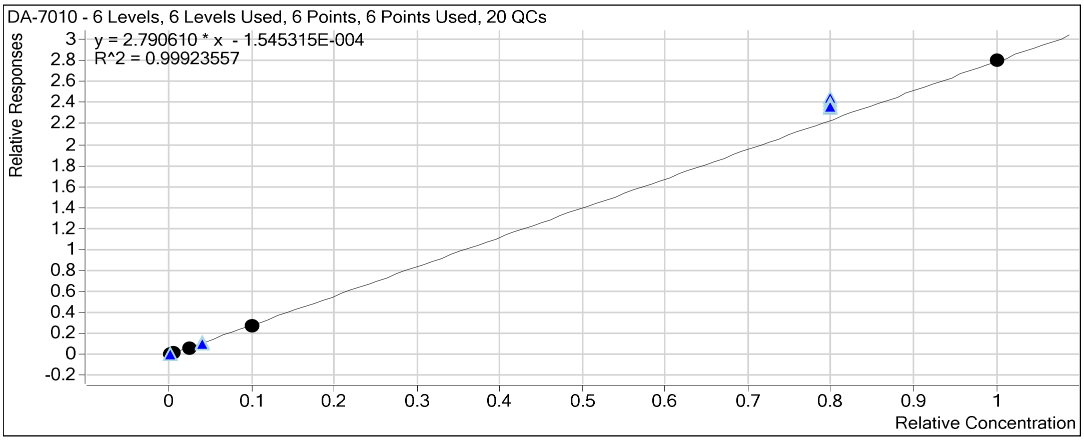

(C)

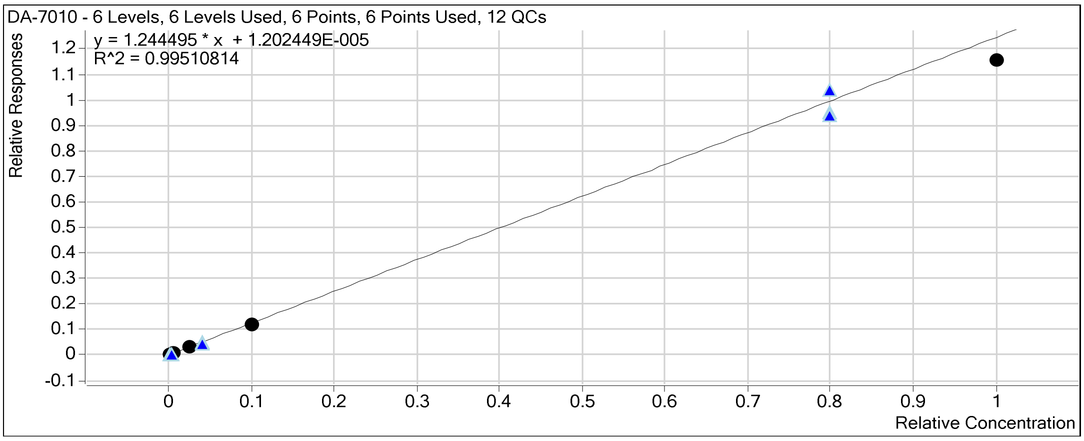

Supplement: Supplementary file 1 [file pharmaceuticals-14-00163-s001.pdf]
